# Neuronal avalanche dynamics indicates different universality classes in neuronal cultures

**DOI:** 10.1038/s41598-018-21730-1

**Published:** 2018-02-21

**Authors:** Mohammad Yaghoubi, Ty de Graaf, Javier G. Orlandi, Fernando Girotto, Michael A. Colicos, Jörn Davidsen

**Affiliations:** 1 Complexity Science Group, Department of Physics and Astronomy, Faculty of Science, University of Calgary, Calgary, AB T2N 1N4 Canada; 2 Department of Physiology & Pharmacology, Faculty of Medicine, and the Hotchkiss Brain Institute, University of Calgary, Calgary, AB T2N 1N4 Canada

## Abstract

Neuronal avalanches have become an ubiquitous tool to describe the activity of large neuronal assemblies. The emergence of scale-free statistics with well-defined exponents has led to the belief that the brain might operate near a critical point. Yet not much is known in terms of how the different exponents arise or how robust they are. Using calcium imaging recordings of dissociated neuronal cultures we show that the exponents are not universal, and that significantly different exponents arise with different culture preparations, leading to the existence of different universality classes. Naturally developing cultures show avalanche statistics consistent with those of a mean-field branching process, however, cultures grown in the presence of folic acid metabolites appear to be in a distinct universality class with significantly different critical exponents. Given the increased synaptic density and number of feedback loops in folate reared cultures, our results suggest that network topology plays a leading role in shaping the avalanche dynamics. We also show that for both types of cultures pronounced correlations exist in the sizes of neuronal avalanches indicating size clustering, being much stronger in folate reared cultures.

## Introduction

The activity of large neuronal assemblies can be described in terms of neuronal avalanches, where groups of consecutive spikes within the whole system are grouped together. These avalanches often show scale-free statistics^1–3^, i.e., the sizes of the cascades have no typical or characteristic scale (other than the system size) and their distribution can be well described by a power law. In many systems, power law behavior is closely related to the concept of criticality, even in nonequilibrium systems^4–6^. Thus, the scale-free characteristics of neuronal avalanches suggests that neuronal assemblies may also operate near a critical point^7,8^.

Despite the broad range of recording techniques which have been used so far to assess scale-free dynamics of neuronal systems, e.g. functional magnetic resonance imaging^9^, electroencephalography (EEG)^10^ and multielectrode arrays (MEA)^1,3^, none of them give a complete view at the single cell level. Although MEA have single-cell and sub-millisecond resolution, their typical inter-electrode spacing and overall size makes a large part of the system inaccessible. Such spatial undersampling can indeed lead to problems in the analysis of neuronal avalanches^11^. To overcome this and also to generate sufficiently high resolution data at the same time, we use optical imaging for the first time to characterize neuronal avalanche statistics in dissociated neuronal cultures. Optical imaging has sufficient temporal resolution to accurately identify and quantify avalanches, and allows recording of all active neurons within the field of view.

We address here the question whether the critical or near-critical behavior of neuronal avalanches can exhibit different universality classes, i.e., whether distinct critical regimes with different power law exponents exist. Indeed, many previous studies found that neuronal avalanches can be mapped to a mean-field branching process to a good approximation^1,12,13^, though more recent experiments showed significant deviations from the mean-field behavior^3^. In some studies on developing cultures and also *in vivo*, deviations of neuronal avalanches from mean-field behavior have also been reported but they were attributed either to sub- or super-critical states^3,8,14,15^. As a specific case study, we focus here on cultured neuronal networks which reared in the presence of the folic acid metabolite 5M4Hfolate. This and other metabolites of folic acid produce an increase in synaptic density and exhibit a dynamics that is much more susceptible to bursting than control cultures^16^. Indeed, we find that while the scale-free dynamics of neuronal avalanches persists in the presence of 5M4Hfolate, the critical exponents are significantly different, suggesting a different universality class. This can be interpreted as a significant change in the underlying structural connectivity related to the emergence of pronounced feedback loops^3,17^. This change in topology is caused by the cultures developing under different conditions, which can have a large impact on the overall dynamics^18^.

We also address the question whether these different universality classes are associated with differences in temporal correlations and memory between neuronal avalanches^19^. We find that the sizes of neuronal avalanches cluster for all different culture preparations such that avalanches tend to be followed by avalanches of similar size; the effect lasting significantly longer in the folate reared cultures.

## Results

### Experimental recordings

**Figure 1:**
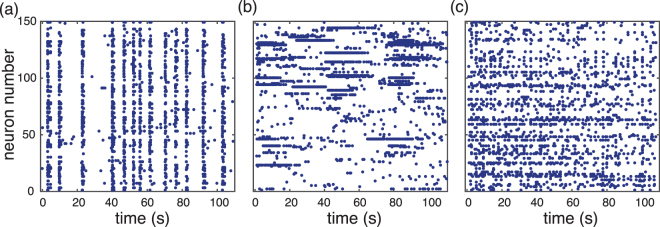
Activity patterns of neuronal cultures. Raster plots of three different cultures showing their characteristic dynamical behaviors over 110s for 150 cells. (**a**) A large number of neurons spike together in a short period of time indicating network bursts, i.e., system-size events (SSE). (**b**) Single-neuron activity with extended bursting periods (type I). (**c**) Asynchronous-irregular activity pattern (type II). Panels (a) and (c) are control cultures and panel (b) is a folate reared culture. However, all these patterns do appear in both preparations of neuronal cultures.

Briefly, experimental data was obtained from networks of dissociated hippocampal neurons co-cultured with glia cells, prepared from newborn P0 Sprague Dawley rats as previously described in ref.^16^ and summarized in Materials and Methods. Folate reared cultures included 50 nM 5M4Hfolate, a concentration based on maternal serum levels of folic acid resulting from supplementation during pregnancy (see ref.^16^). The spontaneous, non-stimulated activity of a square region (approx. 100 to 550 neurons) of the culture was recorded by detecting their fluorescence activity. Individual neurons in the field of view were identified and spikes were inferred from the fluorescence recordings (see Materials and Methods). Both control and folate preparations showed qualitatively similar dynamical behaviors, including system-size events, bursting of single neurons and asynchronous irregular firing as shown in Fig. 1.

#### Neuronal Avalanches

The spike trains are analyzed by identifying possible cascades of induced firings across the observed section of the culture, i.e., neuronal avalanches. Following the standard approach^1,3^, an avalanche is defined here as the largest sequence of consecutive time bins containing spikes in every single time bin. Thus, avalanches are separated by time bins during which none of the neurons in the culture fire. The avalanche duration, *T*, corresponds to the number of time bins. The avalanche size, *S*, is the total number of spikes over the duration of an avalanche. The choice of the size of the time bin has a profound impact on the avalanche definition and its statistics. The bin size is usually chosen relative to signal transmission delays or to the average firing rate^1^. Here, we use the latter approach: We chose our bin size as the overall average inter-spike interval (ISI) across all spikes in a given recording. By construction, this is only meaningful if the average inter-spike intervals do not vary much over the duration of a recording and in the following we focus on such recordings for the avalanche analysis. In addition, the ISIs need to be larger than the inverse of the acquisition speed. Datasets with smaller ISIs would require a higher framerate to accurately separate events. We also verify that our results do not depend on small changes on the selected bin size.

### System-size Events

**Figure 2:**
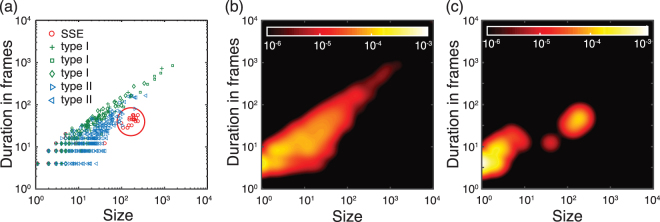
Avalanche statistics in the presence of SSEs. (**a**) The correspondence between avalanche durations and sizes across six different recordings (using the classification from Fig. 1) shows that the dynamical signature of system size events (SSE) is highly regular (large circle). (**b**) Estimate of the average probability density for recordings lacking SSEs. (**c**) Same as in (b), but for a representative recording exhibiting SSEs. The SSEs are clearly separated and distinct from the rest of the events. Colorbars in (b) and (c) show the normalized probability density of events.

Spontaneous activity of young neuronal cultures is often dominated by the presence of network bursts, i.e., quasi-periodic system size events where most of the neurons fire in a short interval^20^. An example can be seen in Fig. 1(a), where the vertical bands of quasi-synchronous firing correspond to these system-size events (SSE). The characteristic spatio-temporal signature of these SSEs is shown in Fig. 2(a), where SSE cluster together in a region of large sizes and short durations, clearly separated from other avalanches. The latter follow a power-law relationship between sizes and duration to a good approximation (see Fig. 2(b)). The deviation of SSEs from this scale-free behavior (see Fig. 2(c)) and indeed their different nature is in line with previous studies^20^ and needs to be taken into account. Simply analyzing SSEs together with the other neuronal avalanches can lead to significant biases in statistical analyses as we show below. Although avalanches can still be defined if one excludes SSEs and just focusses at the neuronal activities between consecutive SSEs, the presence of SSEs restricts the size and duration of neuronal avalanches leading to stronger finite size effects (see Fig. 2(c)). To minimize such finite size effects, we do not include any data sets exhibiting SSEs in the following statistical analyses.

### Critical Avalanches

Historically, branching processes have been used as the conceptual framework for neuronal avalanches^1^; for a branching process at its critical point, there is no characteristic scale and the distribution of both size and duration of avalanches follow a power-law^21^. It should be noted that, even in the case of the validity of the criticality hypothesis for neuronal networks, we are dealing here with a self-organized system which evolves dynamically. This fundamentally differs from standard branching processes, where the system is tuned at a critical point using a control parameter. Interestingly, in the mean-field approximation (corresponding to an infinite dimensional system such that the activity cascade never revisits the same site and feedback loops are absent) almost all self-organized-critical models considered so far, reduce to a critical branching process^6,22^ with known critical exponents and scaling relations. In the following, we look at the distribution of avalanche sizes and durations and the scaling relation between different critical exponents. As scaling theory predicts^4,5^, we have

1 $$f(S)\sim {S}^{-\tau }$$

2 $$f(T)\sim {T}^{-\alpha }$$

3 $$\langle T\rangle (S)\sim {S}^{\gamma }$$

where *f* is the probability distribution function (PDF) of the associated variable. *S* and *T* are the size and duration of the avalanches respectively. The parameters *τ*, *α*, and *γ* are critical exponents of the system and are related through the scaling relation

4 $$\frac{\alpha -1}{\tau -1}=\frac{1}{\gamma }$$

In the mean-field regime, these exponents take on the values *τ* = 1.5, *α* = 2.0, and *γ* = 0.5^3,22^. For comparison, recent experiments on neuronal avalanches in organotypic cultures have found similar exponents for sizes and durations *τ* = 1.6 ± 0.2 and *α* = 1.7 ± 0.2, however *γ* = 0.77 ± 0.03^3^ was significantly different. Similar results are also observed in dissociated neuronal cultures, although the exact values of the exponents depend highly on the methodology^23^. In the following, we present results showing that neuronal avalanches in control cultures show the same critical exponents as the mean-field regime. However, cultures grown in the presence of folic acid show clearly distinct critical exponents.

#### Critical Exponents and Scaling Relation

To test the hypothesis of critical avalanches, we measure the critical exponents defined in Eqs (1–3) by different means. For *τ* and *α*, we typically use a maximum likelihood estimation (MLE) and goodness of fit test. We exhaustively investigate any possible dependence on the specific fitting interval to establish: (i) the existence and statistical significance of power-law behavior, and (ii) the robustness of the estimated exponents^24,25^ (see Materials and Methods for details). This is particularly important due to the presence of finite size and finite sample effects. For *γ*, we use least squares fitting instead (see Materials and Methods for details).

**Figure 3:**
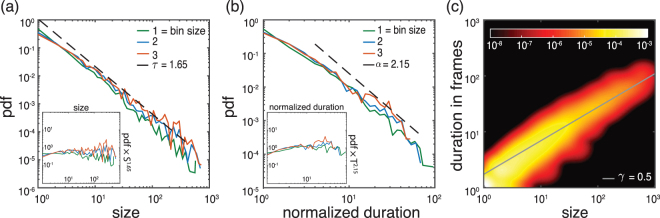
Exponent analysis for control cultures. (**a**)–(**c**) show the probability density function (PDF) of avalanche sizes, avalanche durations, as well as the joint PDF for size and duration, respectively, for a characteristic control culture. For this recording, the overall average inter-spike interval (ISI) is 32 ms and, thus, comparable to our temporal resolution/frame rate (30 ms). Throughout the whole article, we keep the convention that the temporal bin size of 1 corresponds to 30 ms and that the normalized duration corresponds to the duration of an avalanche measured in units of the chosen bin size (multiples of 30 ms). The two subpanels in (a) and (b) are the rescaled PDFs (i.e. multiplied by *S*^*τ*^, or *T*^*α*^ for size and duration, respectively). Maximum likelihood estimation for the size distribution and the duration distribution and least squares fitting for the joint PDF give *τ*_*control*_ = 1.65 ± 0.1, *α*_*control*_ = 2.15 ± 0.2 and *γ*_*control*_ = 0.5 ± 0.1 for bin size 1. These exponents are within range of what is expected in the mean-field limit and are consistent with the scaling relation (4). See Materials and Methods for an extended analysis.

**Figure 4:**
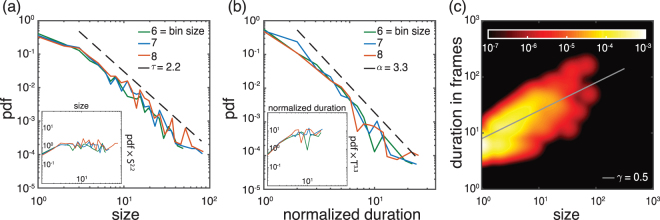
Exponent analysis for folate reared cultures. Same as in Fig. 3 but for a characteristic folate reared culture. For this recording, the ISI is 220 ms corresponding to a bin size of about 7. The critical exponents are *τ*_*folate*_ = 2.2 ± 0.2, *α*_*folate*_ = 3.3 ± 0.4 and *γ*_*folate*_ = 0.5 ± 0.1 for bin size 7. The values of *τ*_*folate*_ and *α*_*folate*_ significantly differ from those for control cultures (see Fig. 3) and what is predicted by mean-field theory. Yet, they are still consistent with the scaling relation (4). See Materials and Methods for an extended analysis.

As an example, Figs 3 and 4 show the different PDFs of the neuronal avalanches for a folate reared culture and a control culture, respectively. Starting with the avalanche size distribution for the control cultures, *τ*_*control*_ exhibits a slight dependence on the bin size (see also Materials and Methods). However, the observed exponents are consistent with the one expected in the mean-field limit. Specifically, for a bin size of 1 (corresponding to 30 ms) *τ*_*control*_ = 1.65 ± 0.1. On the other hand, the exponent found for folate reared cultures is significantly different, *τ*_*folate*_ = 2.2 ± 0.2. This value is robust with respect to variations in bin size (see also Materials and Methods). A similar behavior holds for the avalanche durations. Figure 4 shows that *α*_*control*_ = 2.15 ± 0.2, *γ*_*control*_ = 0.5 ± 0.1 for the control culture, while Fig. 3 shows that *α*_*folate*_ = 3.3 ± 0.4, *γ*_*folate*_ = 0.5 ± 0.1 for the folate reared culture. To establish the consistency of the obtained exponents with the scaling relation (4), we need to compare the directly estimated values with those predicted by the other two exponents using the scaling relation. Indeed, given the statistical uncertainties both sets of exponents are consistent with the scaling relation.

**Figure 5:**
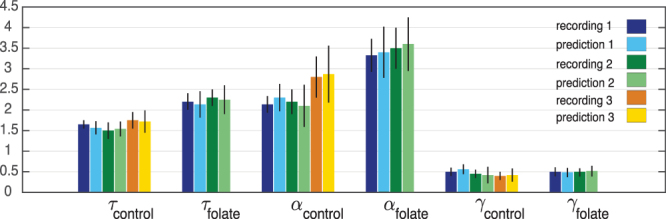
Critical exponents. All exponents for both control and folate cases over different recordings. Different colors represent different recordings. For each color (blue, green, orange), the bright one is obtained by direct measurement (see Materials & Methods) and the pale color represents the exponent predicted by scaling theory (Equation (4)) using the directly estimated values of the other two exponents. The exponents are fairly robust over different recordings. For both conditions, the scaling relation holds, while we have significantly different critical exponents *α* and *τ* for the two different conditions. Error bars of the measured exponents correspond to 95% confidence intervals (see Materials and Methods and Figs 8–10 for an extended analysis) and the error bars of the predicted exponents are obtained by using linear error propagation.

As Fig. 5 shows, our findings are not specific to the two examples shown in Figs 3 and 4 but hold for all recordings we analyzed. Specifically, as Fig. 5 shows, the folate case exhibits significant deviations from the mean–field behavior, while the exponents for the control case are largely statistically indistinguishable from the mean-field values as also reported in previous studies^3^. Taking error bars into account, we also see that the measured exponents in the folate case are the same as the predicted ones based on the scaling relation. Having this consistency and given the fact that the hypothesis of a power-law behavior over extended ranges cannot be rejected at any reasonable confidence level for the various variables (see Materials & Methods) suggests that the neuronal network developed in the presence of folic acid may operate close to a critical point. Yet, this critical point seems to be totally different from the critical point observed in critical branching process, indicating a different universality class.

### Size correlations and clustering

To establish whether there is any memory in the size distribution of the neuronal avalanches, we test the null hypothesis that the sizes of the *i*th avalanche and the (*i* + *j*)th avalanche are independent. Specifically, we focus on the size ratio *S*_*i*+*j*_/*S*_*i*_ due to the wide range of avalanche sizes. If event sizes were independent, the statistical distribution of the size ratio should not deviate significantly from the case where avalanche sizes are randomly selected. This can be captured by the difference in the cumulative probabilities defined as $\delta P({S}_{i+j}/{S}_{i} < \lambda )=P({S}_{i+j}/{S}_{i} < \lambda )-P({S}_{i+j}^{\ast }/{S}_{i}^{\ast } < \lambda )$, which has been proven helpful in the context of earthquakes^26–28^. Here, S^*^ refers to surrogate data obtained by randomly reshuffling the order of the avalanche sizes. The ensemble of surrogate data also allows one to estimate the statistical uncertainties in *δP*(*S*_*i*+*j*_/*S*_*i*_ < *λ*).

**Figure 6:**
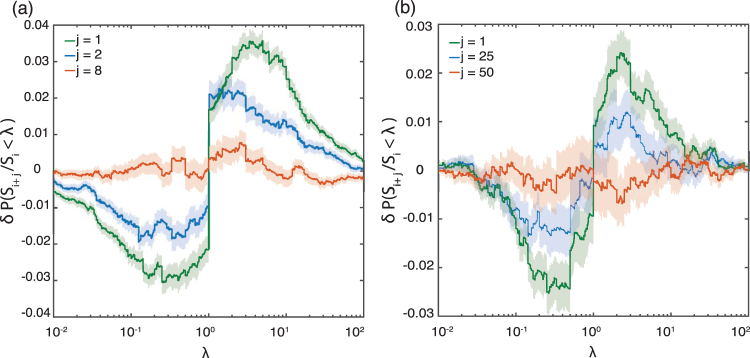
Size correlations. The quantity *δP*(*S*_*i* + *j*_/*S*_*i*_ < *λ*) as a function of *λ* is depicted (see text for details). Qualitatively, the same behaviour is observed for both control (left) and folate reared (right) cultures corresponding to the recordings discussed in Figs 3 and 4, respectively. Shaded area show ± 1 standard deviation of the measure. Significant non-zero values of *δP*(*S*_*i* + *j*_/*S*_*i*_ < *λ*) indicate correlations in the sequence of avalanche sizes. These correlations are particularly pronounced for *j* = 1 but disappear for larger *j*. Red curve shows the first *j* value at which correlations completely disappear (*j* = 8 for control and *j* = 50 for folate reared cultures). The positive slope of *δP*(*S*_*i* + *j*_/*S*_*i*_ < *λ*) around *λ* = 1 indicates that it is more likely that an event is followed by events similar in size, compared to what would be expected if event sizes were independent.

The corresponding findings for different neuronal cultures are shown in Fig. 6. In all cases, one can reject the null hypothesis of independent avalanche sizes for *j* = 1 at high confidence levels. Instead, the behavior of *δP*(*S*_*i*+*j*_/*S*_*i*_ < *λ*) as a function of *λ* indicates that the probability that *S*_*i*+*j*_/*S*_*i*_ falls between 3 × 10^−1^ and 3 is about 5% higher for the folate case compared to what is expected for independent avalanche sizes. This implies that neuronal avalanches tend to cluster in terms of their sizes: Avalanches are followed by avalanches of similar size — similar meaning here a variation by a factor between about 1/3 and 3. In the control case, this probability is about 7%, comparable with observations for cortex slice cultures under non-driven conditions^19^. For both control and folate reared cultures, this probability decreases with increasing *j* such that for sufficiently large values of *j* (specifically for *j* ≥ 50 for folate reared and for *j* ≥ 8 for control cultures) one cannot reject the null hypothesis anymore. This results shows that temporal correlations of avalanche sizes in folate reared cultures lasts for a longer time in comparison with control cultures. These results are robust with regard to the choice of bin size and also across the different recordings.

## Discussion and Conclusions

The analysis of the spontaneous activity of dissociated neuronal cultures grown in control conditions and in the presence of folic acid reveals avalanche-like dynamics consistent with critical or near-critical behavior. The set of critical exponents, however, shows significantly different exponents between the two conditions. In control cultures, the measured scaling exponents, *τ*_*control*_ = 1.65 ± 0.1, *α*_*control*_ = 2.15 ± 0.2, *γ*_*control*_ = 0.5 ± 0.1, are consistent with scaling theory and with previous reports (see, for example, refs^1,14,15,29^). In the case of folate reared cultures, however, the neuronal avalanches exhibit significantly different critical exponents from the control case, namely *τ*_*folate*_ = 2.2 ± 0.2, *α*_*folate*_ = 3.3 ± 0.4, *γ*_*folate*_ = 0.5 ± 0.1. The relationship between the exponents is still consistent with the scaling relation (4) and, thus, scaling theory. All these observations together indicate that these two dynamical systems belong to different universality classes. Moreover, significant deviations from mean–field theory suggest that in contrast to the control case, avalanches in folate reared cultures arise from a much richer dynamics and interactions such that information propagation as captured by neuronal avalanches cannot be well approximated as a simple branching process without feedback loops and/or memory. Specifically, it suggests that the structural connectivity within the folate reared cultures plays a much more important role compared to the control cultures, similar to what has been proposed in other cases^3^. This is especially interesting given that there is evidence that the presence of folic acid alters the structural connectivity of developing neuronal systems^16^. This is related to the observation that folate reared neuronal cultures are more susceptible to seizure–like behavior when the system experiences a system–size stimulus and might have its origin in the changes in the firing behavior of individual nerve cells induced by folic acid^16^. Having a larger exponent *τ* characterizing the size distribution of folate cultures indicates that the system has self–organized in such a way that it is less likely to spontaneously generate large neuronal avalanches compared to the control case. Thus, this self-organized state seems to minimize the occurrence of spontaneous seizure–like behavior arising from neuronal avalanches. To verify the role of the structural connectivity in the case of folate reared cultures and beyond, it is highly desirable to study the interplay between structure and dynamics explicitly. Yet, estimating the structural connectivity including inhibitory connections from limited spike train data as in our case here remains a challenge for the future.

## Materials and Methods

### Neuronal Cultures & Data Acquisition

All animal care protocols were approved by the University of Calgary’s Animal Care and Use Committee in accordance with the Canadian Council on Animal Care guidelines. All experimental procedures were carried out in accordance with these approved guidelines. Experimental data was obtained from networks of dissociated hippocampal neurons co-cultured with glia cells, prepared from newborn P0 Sprague Dawley rats as previously described in ref.^16^. Growth medium consisted of Basal Medium Eagle supplemented with B27 and decreasing amounts of Fetal Bovine Serum (5% → 0% over 2 weeks), and was changed bi-weekly by replacing 50% of the volume with fresh medium. Folate reared cultures included 50 nM 5M4Hfolate, a concentration based on maternal serum levels of folic acid resulting from supplementation during pregnancy (see ref.^16^).

A square region of 500 × 500 *μm*^2^ (approx. 100 to 200 neurons) of the culture was recorded by detecting their fluorescence activity under low magnification with a CCD camera of high sensitivity (Hamamatsu ORCA-Flash4). Fluo-4, a fluorescent calcium dye, was added to the cultures 20 minutes before imaging to serve as an indicator of neuronal firing. Each culture was recorded for a maximum of 20 minutes per region to avoid photo-damage and bleaching. Acquisition speed was 33 frames per second, resulting in 30 ms temporal resolution. All recordings were taken between 17 and 26 days *in vitro*. The specific details of all recordings are given in Table (1).

**Table 1:** Information aboute the analyzed recordings.

	culture age	recording time	no. of cells	culture average firing rate	neuronal firing rates’ coefficient of variation	ISI	no. of avalanches in the fit range for *τ*
**folate**							
recording 1	17 days	15 min	174	4.35 spike/s	0.62	220 ms	425
recording 2	19 days	20 min	228	7.29 spike/s	0.89	137 ms	1400
**control**							
recording 1	17 days	20 min	80	34 spike/s	0.70	32 ms	4769
recording 2	26 days	15 min	100	13 spikes/s	1.0	75 ms	530
recording 3	26 days	20 min	92	6.5 spikes/s	0.67	155 ms	495

### Cell Detection and Spike Inference

**Figure 7:**
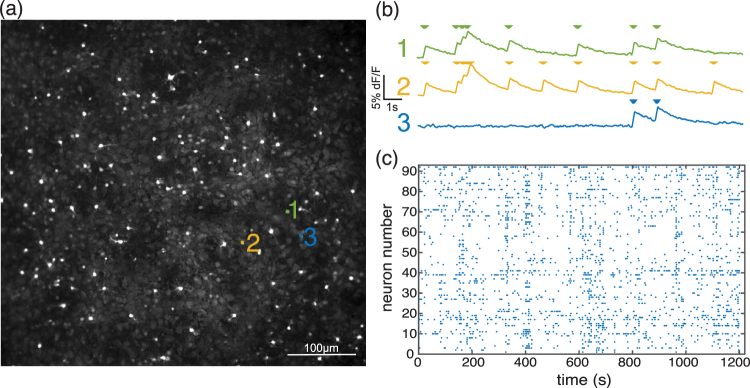
(**a**) Average of fluorescence recording, (**b**) Calcium trace detected spikes of three neurons, and (**c**) raster plot of one of the recordings are presented.

For cell detection and spike inference we largely used the same methodology as in ref.^16^, see Fig. 7. In brief, cells positions were automatically detected from the averaged fluorescence recording, which was obtained by temporally averaging every frame of the first 2 minutes of the recording and applying a two dimensional median filter with a block size of 32 pixels to remove shot noise. Cell contours were then obtained from the following morphological operations: the average image background was obtained by morphologically opening the average frame with a disk 16 pixels wide. Then the average frame was binarized by removing its background and performing another morphological opening with a disk 1 pixel wide and thresholding the result. Any connected component with an overall size bigger than 10 pixels was considered a putative cell. Putative cells with a solidity below 0.9 were split into smaller cells by further thresholding the original cell image against its median and identifying new connected components. Any new cell smaller than 10 pixels was discarded.

The fluorescence signal for each cell was obtained by spatially averaging across all cell’s pixels. The temporal signal was then smoothed via a square filter running average with size 0.2 s. Baseline fluorescence was computed with a smoothing spline and posteriorly subtracted. Traces were further normalized to Δ*F*/*F*_0_, where *F*_0_ was computed as the 5th lower percentile of the original fluorescence signal. Traces that corresponded to glial cells or silent cells were discarded (via a pattern-matching algorithm). Spikes in the remaining traces were inferred via the OASIS algorithm^30^. All fluorescence data analysis was performed with NETCAL^31^. Note that the observed ISI values (see Table (1)) are substantially larger than the ones one observes if the experimental recordings are not done at room temperature but at about 37 °C^14,29^, which typically leads to higher firing rates.

### Maximum Likelihood Estimation

The likelihood function for a power law distribution characterized by an exponent *γ* for a discrete data set containing *n* values is defined as:

5 $$L=P(x|\gamma )=\prod _{i=1}^{n}P({x}_{i}).$$

The value of *γ* that best fits the data is the *γ* that maximizes *L*, denoted by *γ*_*max*_^32^. It can be shown that ${[-\frac{{\partial }^{2}\mathrm{ln}L(\gamma ={\gamma }_{{\rm{\max }}})}{{\partial }^{2}\gamma }]}^{-\frac{1}{2}}$ can be used as an estimator of the standard deviation of the estimated exponent^33^. We choose 2*σ* as the uncertainty of the obtained critical exponents throughout the article.

### Goodness of Fit Test

The Kolmogorov-Smirnov (KS) statistic quantifies the distance between two probability distributions^34^, which is defined as the maximum distance between the cumulative distribution functions (CDFs) of the two distributions (here data and fitted model):

6 $${d}_{e}=\,{\rm{\max }}|{S}_{e}(x)-{P}_{e}(x)|,\quad {X}_{low}\le x\le {X}_{high}.$$

Here, *S*_*e*_(*x*) is the CDF of the empirical data and *P*_*e*_(*x*) is the CDF corresponding to the fitted model. The *p*-value is the probability to observe a KS value bigger than *d*_*e*_ for synthetic data generated by the fitted model and provides a measure whether it is likely that the empirical data do indeed follow the fitted model. One can show that its value can be calculated from the following theoretical expression^25^:

7 $$p-{\rm{value}}=2\sum _{i=1}^{\infty }{(-1)}^{i-1}\exp [-2{i}^{2}{({d}_{e}\sqrt{n}+0.12{d}_{e}+0.11{d}_{e}/\sqrt{n})}^{2}],$$

where *n* is the number of discrete values observed in the data set.

Although, it is known that the given analytical equation (Equation (7)) can overestimate the *p*-value^25^, it nevertheless provides a good measure to compare different intervals at a low computational cost. After picking the extended interval exhibiting a *p*-value higher than the other intervals based on Equation (7), we calculate a more accurate *p*-value using the alternative, surrogate-based method described in ref.^25^. For the selected intervals across the different recordings, we found that Eq. (7) gives a *p*-value close to one, while the alternative method^25^ gives a smaller value but still bigger than 0.2. This indicates that the power law hypothesis for the size and duration distributions in neuronal avalanche dynamics cannot be rejected with any reasonable confidence.

Note that we have also tested individual recordings against alternative distributions, such as exponential, Yule, Poisson and log-normal distributions. In all cases, the power-law hypothesis showed a higher *p*-value than the other distributions except for Yule distribution. In control cultures, the *p*-values associated with the avalanche sizes for power-law and Yule distributions were 0.2 and 0.3, respectively. For folate cultures, we obtained 0.6 and 0.7 for power-law and Yule distributions, respectively. The *p*-value for the remaining distributions were essentially zero. Although our data is also compatible with the Yule distribution — and in some regards might be a slightly better fit due to its discrete nature — we decided to focus on the power-law distribution as in other comparable studies since the asymptotic behavior is the same in both cases.

### Estimation of the Critical Exponents

**Figure 8:**
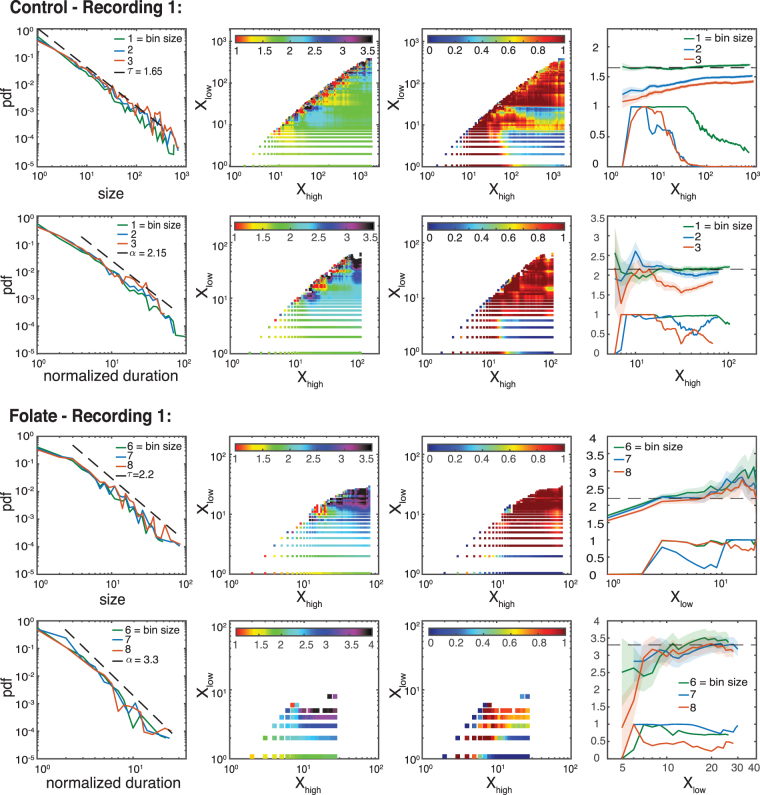
Extended analysis of the critical behavior for the recordings shown in Figs 3 and 4. All rows show the same line of analysis for different critical exponents and recordings. First column: PDF of events (size or normalized duration). Second column: Estimation of the critical exponent for different lower (*X*_*low*_) and higher (*X*_*high*_) cut-off values using the bin size closest to the ISI. Third column: Associated *p*-values. Red areas suggest plausibility of scale-free hypothesis. Forth column: Dependence of critical exponents (the upper lines with errorbar) and *p*-values (the lower lines without errorbar) on the selected interval once we have fixed one side of the interval (for control and folate *X*_*low*_ and *X*_*high*_ are fixed, respectively). Robust estimates of the exponents are characterized by high *p*-values and an extended regime over which their estimated value does not change significantly. We find: *τ*_*control*_ = 1.65 ± 0.1, *τ*_*folate*_ = 2.2 ± 0.2, *α*_*control*_ = 2.15 ± 0.2, and *α*_*folate*_ = 3.3 ± 0.4 (for the full list of critical exponents over different recordings see Fig. 5).

**Figure 9:**
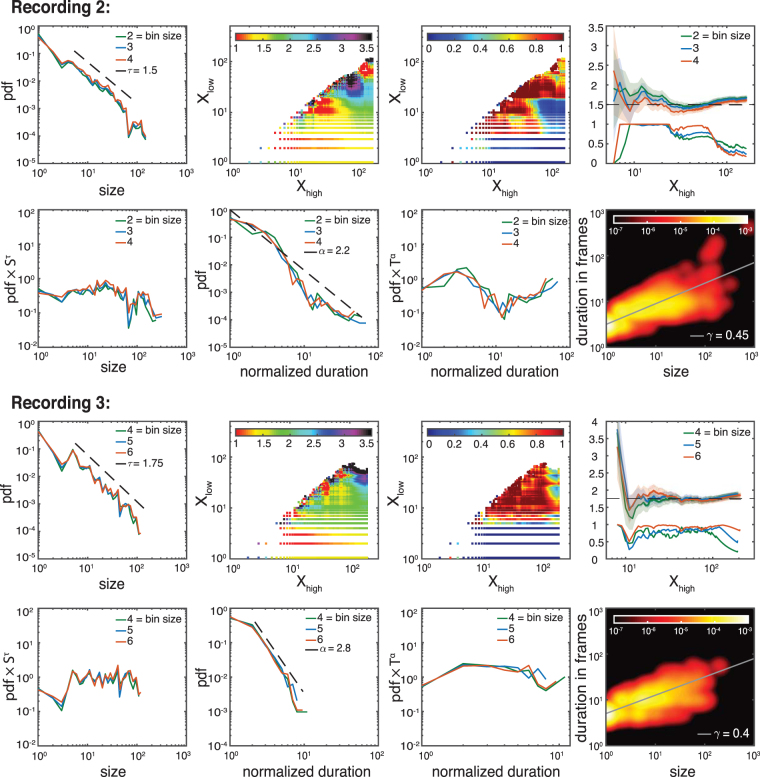
Exponent analysis of other control recordings (recordings 2 and 3). Similar to Fig. 8. Due to the limited range of the distribution function for avalanche durations — as also observed in other studies, e.g. ref.^1^ — we were not able to use the MLE method in a reliable way. Instead, we use the rescaled PDFs as a graphical method to get a rough estimate of the best possible fit and range. The results are comparable to the ones obtained by least square method. As before, we use a least square method to estimate *γ* over the ranges determined by our estimation procedure for *τ* and *α* and give 2 *σ* error bars. We find: *τ*_*control*_ = 1.5 ± 0.2,1.75 ± 0.2, *α*_*control*_ = 2.2 ± 0.3,2.8 ± 0.5, *γ*_*control*_ = 0.45 ± 0.1,0.4 ± 0.1 (for the full list of critical exponents over different recordings see Fig. 5).

**Figure 10:**
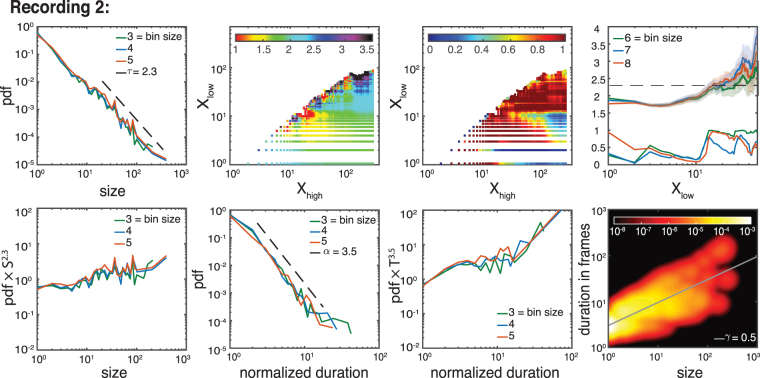
Exponent analysis of other folate case (recording 2). As in Fig. 9. The estimated exponents are: *τ*_*folate*_ = 2.3 ± 0.2, *α*_*folate*_ = 3.5 ± 0.5 and *γ*_*folate*_ = 0.5 ± 0.1 (for the full list of critical exponents over different recordings see Fig. 5).

Using maximum likelihood estimation we calculate the critical exponents for all possible intervals and plot them in an exponent color-map (see Figs 8–10). The existence of a flat plateau with homogeneous color in the exponent map provides evidence of scale free behavior over that range. Additionally, to test the plausibility of the power-law hypothesis for those intervals, we provide the goodness-of-fit based on Equation (7). Its *p*-value signifies when the power-law hypothesis can be rejected at high confidence, usually for *p* < 0.1^35^. The coexistence of a flat plateau in the exponent color map and a large *p*-value for a wide range of event sizes is compatible with the existence of scale free behavior. Note that deviations from a pure power-law behavior for small arguments and large arguments are typically expected, for example, due to finite size/finite sample effects.
